# A Mixed-Method Analytical Cross-Sectional Research on Participation-Centered Learning Behaviors for Adolescent and Adult Learners Utilizing a Validated Learning Behavior Questionnaire

**DOI:** 10.7759/cureus.59178

**Published:** 2024-04-27

**Authors:** Nirupama Bhise, Vedprakash Mishra, Sweta Pisulkar, Sharayu Nimonkar, Chinmayee Dahihandekar, Vikram Belkhode

**Affiliations:** 1 Management, Jawaharlal Nehru Medical College, Datta Meghe Institute of Higher Education and Research, Wardha, IND; 2 Physiology, Jawaharlal Nehru Medical College, Datta Meghe Institute of Higher Education and Research, Wardha, IND; 3 Prosthodontics, Sharad Pawar Dental College and Hospital, Datta Meghe Institute of Higher Education and Research, Wardha, IND

**Keywords:** participation-centred learning, behavior, adult learner, adolescent learner, self-monitoring checklist

## Abstract

Background

In their academic lives, students progress from the stage of primary learning to the stage of adolescent learning and then to the stage of adult learning. At every step of learning, learners display particular learning habits, which must be mapped out to maximize learning.

Objectives

The objective of the present study is the evaluation of the participation-centered learning behaviors among adolescent and adult learners employing a validated learning behavior questionnaire.

Material and methods

This was a cross-sectional research. A total of 944 participants were in the study, including 456 adolescents from English-medium schools (aged 11 to 16) and 488 adults from a health professional institute ( aged 18 to 23 years). The validated learning behavior questionnaire, which study participants rated on a scale of 0, 1, and 2, served as the quantitative component. The focus group discussion that was held for a group of adult and teenage students comprised the study's qualitative component. Using STATA-14 software (StataCorp, College Station, United States), all of the responses were tallied and statistically examined.

Results

The mean scores of participation-centered learning behaviors were significantly higher in adult learners than in teenage learners. The findings of the qualitative component analyzed were consistent with the findings of the learning behavior questionnaire analysis.

Conclusion

The study's self-monitoring checklist may aid in the evaluation of learning behaviors and make it simpler for adult and adolescent learners to establish excellent learning habits.

## Introduction

Learning is a lifelong process that begins at birth and lasts until a person passes away. Learning entails taking in information with all five senses, analyzing it, and assimilation the information in the human brain according to “willingness, values, and a person's beliefs” [1]. The 20th century saw the beginning of the investigation of learning from a scientific perspective. One of the first academics to advocate for his concept of comprehensive education to address the rapidly changing business and society was John Dewey (1938-1997) [2]. Theories of learning guide the learning process in every individual. These age-specific learning theories are crucial to understanding how a learner learns. According to Khalil and Elkhider's study, the antecedents to learning theories emerged as intellectual notions such as “rationalism and empiricism” and were summarized in psychological research on "functionalism and structuralism" [3]. The theories of “behaviorism, cognitivism, and constructivism” are some examples of “theories of learning.” Behaviorist theories highlight the importance of the connection between the stimulus and the response and describe learning as a response to natural stimuli. It further argues that answers are more likely to be repeated when they are followed by reinforcements. The cognitive theories of learning place a strong emphasis on knowledge acquisition and the brain's processing of this knowledge as the fundamental elements of learning. The learner who is an active participant views information acquisition as a mental process involving coding and structure [4]. The constructivism philosophy promotes the idea that a learner acquires knowledge via their experiences and interactions. Learning takes precedence over teaching along the continuum of learning theories, from a “passive student” to an “active learner,” and from the “passive” student to an “active learner,” and transfer from the passive transition of information to the learner to the learner's active application of knowledge [5].

In terms of learning behaviors, “self-regulation and monitoring,” especially during the adolescent phase, can help learners make the transition from adolescent to adult learners. Therefore, developing a self-monitoring checklist will greatly help the learner to keep track of his or her learning behaviors [6]. Teachers, facilitators, and parents can evaluate a learner's conduct using a variety of scales. For students with special educational requirements, self-monitoring scales are available; however, the demands of students generally regarding “self-monitoring of the learning behaviors” have been disregarded, and no measurable parameters are provided for the learner himself/herself [7]. In response to the study's question, "Will an appropriate self-monitoring checklist developed for self-assessment of learning behaviors be helpful in both adolescent and adult learners?" a self-monitoring checklist predicated on learning behaviors was developed to analyze "participant-centered learning behaviors."

## Materials and methods

Ethical consideration

Students from grades 6th to 10th with the ages ranging between 11 to 16 years at an “English medium with Central Board of Secondary Education (CBSE) board school,” as well as pupils from first to fourth year and interns from 18 to 23 years of age at “health professional institutions,” took part in the study. The trial was done from March 2016 to February 2020. The study was conducted at Sharad Pawar Dental College and Hospital, Wardha, India. The current study received approval from the “Institutional Ethics Committee of Datta Meghe Institute of Medical Sciences (Deemed to Be University)” with the reference number DMIMS (DU)/IEC/2015-16/1753. Before the study, informed consent and assent forms were sought from the guardians of the teenage learners and also from the adult learners in a “health professional institute.” The motivation for the study project was assessed for both teenage and adult learners. Participants were informed that their information would be kept private.

Sample size

A N Master V.2.0 was the program used to calculate the sample size. According to the calculation, the sample size was 944, among which 456 were adolescent learners and 488 were adult learners. After screening as research participants, the 944 respondents who were enrolled in the current study reacted satisfactorily.

Study population

Adolescent learners included were from class VI to grade X at an English medium with CBSE board school between the ages of 11 and 16 years from both femininities. Adult learners include first-year interns at a “health professional institute” between the ages of 18 and 23 (both femininity). Exclusion criteria for adolescent students in class VI to X in an ”English medium with CBSE board school” and adult students in their first year as interns at a “health professional institute” who were not present for three visits in a row for questionnaire sessions.

Data collection

A learning behavior questionnaire was employed in the current study. The questionnaire was validated and developed. The questionnaire was first designed with three learning domains in mind namely cognitive, emotional, and interpersonal, and featured 15 items to be assessed on a two-point rating system. The above questionnaire was given to “behavioral psychology and teaching-learning” specialists for their thoughts, ideas, and recommendations. In light of the ideas obtained, a questionnaire was developed and distributed to 20 grade VIII students including teenage students and 20 fourth-semester medical students signifying adult students, with the same number of male and female participants. Furthermore, the learning behavior questionnaire was validated for relevance and application on a “stratified sample” of 10 students from every grade of both “adolescent and adult learners.” For validation, a team of four experts in the field from the School for Higher Education and Research Unit was appointed and a meeting was conducted. The panel of experts scrutinized the questionnaire and after their consent, the scoring was evaluated. The scoring above the average gave a validation report to the questionnaire. Aside from the above objective claims, the learning behavior questionnaire included a “qualitative component” based on the learners' perceptions, in which they had to rank the declarations linked to learning behaviors in preference order. The study participants were given the validated version of the learning behavior questionnaire. The replies were analyzed to identify and compare learning behaviors in both research groups. The validated learning behavior questionnaire includes 15 items regarding behaviors related to task start, continuance, and completion under the areas of “goal setting, motivation, responsibility, and self-discipline.” The validated learning behavior questionnaire was given to all students in the “English-medium CBSE board school” in grades 6 through 10, as well as to adult learners in their first, second, third, and fourth years, and interns from the “health professional institute,” in accordance with the inclusion criteria.

Statistical analysis

The “qualitative component” included two “focus group discussions” (FGDs), one for teenage learners and one for adult learners. The FGD members were chosen using computer-generated randomness. Through this method, two representatives from grades VI to X, as well as first-year interns, were chosen from the group of teenage learners and adult learners. The responses of all teenage and adult learners to the learning behavior questionnaire were totaled and statistically analyzed with “STATA-14 software” (StataCorp, College Station, United States). To assess intra- and inter-group variation and significance, paired and “chi-square tests” were utilized. Random selection of the study subject was done and differences between the pairings had a roughly normal distribution; hence, a paired t-test was used. Since the data obtained was analyzed as categorical data, chi-square tests were used.

## Results

In adult learners, significant values were found for quantitative components of participation-centered learning behaviors such as goal-setting, motivation, responsibility, and self-discipline. In adolescent and adult learners, the number of participants who rated various items on the participation (Pa) scale was 0, 1, and 2. The table above shows the number of participants in adolescent and adult learners who rated various items on the participation-centered learning behavior scale as 0, 1, or 2. The highest grade, 2 for "I learn well when I study in a group or do group activities" (Pa3), "Group activities like quizzes, role plays, debates, talks, and seminars help me learn independently" (Pa5), "Relevant projects and assignments, if given for a difficult topic, make it easy to understand" (Pa6), "I ask others for help when I do not understand something" (Pa7), "I help my friends in difficulties if they ask me for help" (Pa8), "Discussing a topic with my friend helps me to understand it thoroughly" (Pa9), "In case I do not understand the use of" (Pa10), "The teaching and non-teaching personnel contribute to the establishment of a meaningful learning environment" (Pa11), "I make an effort to form positive relationships with my instructor and students" (Pa12), "I know how my instructor thinks about me and what she expects from me" (Pa13), "I regard my peers' self-esteem as much as I value mine" (Pa14), "Equal chances for learning are offered in the classroom for all pupils" (Pa15), "My connections do not interfere with the obligations assigned to me" (Pa16), "If I am having difficulty with a writing task, I seek assistance from accessible resources such as peers, teachers, learning centers, or tutoring centers" (Pa17). When compared to teenage learners, adult learners had considerably more items (p < 0.05). The mean difference between participation-centered learning practices in adult learners was shown to be substantial when compared to teenage learners in the adolescent learner category, the participation-centered learning behaviors received mean scores of 10.43 (2.98) and 10.67 (2.64) for males and girls, respectively. Similarly, in the adult learners category, male students had a mean score of 12.18 (3.51) and female students had a score of 12.47 (3.32). The difference in mean scores for participation-centered learning is statistically insignificant at the 5% level of significance in both adolescents and adults, at p = 0.368 and p = 0.415, respectively. The qualitative findings from the FGDs for both teenage and adult learners show the learners' views and perspectives. The difference in mean scores for participation-centered learning and the comparison of responses from teenage and adult learners is shown in Tables 1, 2.

**Table 1:** Comparison of responses of adolescent and adult learners Coef.: coefficient of variation; Std. Err.: standard error; T: t-test, p-value of significance; SD: standard deviation

S.No	Learning behavior	Adolescent (n = 488) mean ± SD	Adult (n = 456) mean ± SD	Coef. (95% confidence interval)	Std. Err.	T	p-value
Participation-centered learning behaviors							
1	Engagement	1.76 ± 0.91	1.86 ± 0.95	0.17 (0.07-0.26)	0.001	3.26	0.001
2	Collaboration	5.54 ± 1.78	6.42 ± 2.06	1.60 (1.29-1.91)	0.154	10.34	0.000
3	Communication	6.48 ± 1.87	7.32 ± 2.10	0.44 (0.31-0.56)	0.065	6.68	0.000
4	Responsiveness	1.36 ± 0.64	1.58 ± 0.62	0.23 (0.14-0.30)	0.041	5.45	0.000

**Table 2:** Comparison of responses of adolescent and adult learners

		Adolescent	Adult
Collaboration and communication	Dissimilarities	Self-studies help in better understanding and learning. These involve independent learning behavior.	Group studies help in better understanding and learning. These involve the learning behaviors of collaboration and communication.
Communication		Need the help of some elderly person to solve academic and personal problems.	Believe that only academic problems can be solved by interaction with peers.
Collaboration and communication	Similarities	Assignments help in better understanding and learning. These involve the learning behaviors of collaboration and communication.	
Positive relations		Positive environment and relationships help in effective learning.	

## Discussion

The learning behavior that a student exhibits in the classroom frequently determines how well they succeed academically. Studies on learning theories and behaviors have employed a variety of descriptors to describe learning behaviors, according to the research [8]. Due to the complexity of learning behaviors and the analysis that surrounds them, there is a dearth of literature on the subject. As a result, it is necessary to examine the concept of learning behavior. Powell and Tod conducted a systematic review earlier in 2004 in which learning behaviors were categorized as "product-centered," "participation-centered," and "person-centered learning behaviors" founded on the characteristics that researchers employed in 46 different studies [1]. The close connection between "curriculum" "others" and "self," as a foundation for efficient learning was highlighted by this systematic review. With evolving patterns and educational regulations, there is a need to promote “self-monitoring of learning” behaviors to inspire students to adopt positive learning habits that will enable them to implement the necessary corrective actions to enhance their performance. As a result, a self-monitoring questionnaire was created with adult and adolescent learners in mind.

There is a dearth of information regarding a cumulative assessment of the many learning behavior factors. There is research where only the impact and application of specific learning behaviors are assessed [9]. Therefore, the current study was designed to include data and analysis together with the results to meet the study's translatory component. The participation-centered learning behaviors deal with the learning behaviors of "Participation," "Engagement," "Collaboration," "Communication" and "Responsiveness." These learning behaviors impact the social context of the classroom influencing the collaborative learning of the learners involving the wholehearted participation and full-time engagement of the learners with the help of appropriate communication. In an article published in 2019, participation is said to be a learning behavior and a strategy that is immensely effective in the teaching and learning process. The findings of the study conclude that participation in group learning requires mutual respect among team members, responsibility toward the group activity, a conducive environment, and leadership [10]. Participation in a class is often treated as the responsibility of the learners, and responses received in the class are treated as engagement. It is said that the best learning takes place when learners are actively participating in the process of learning [11].

The current study’s findings indicate that the participation-centered learning behaviors of "Participation," "Engagement," "Collaboration," "Communication" and "Responsiveness" have statistically significant values (p = 0.00), in adult learners as opposed to learners in their teens.

This is probably due to the fact that these behaviors develop with age and the maturation of the learner. As per Thijs and Verkuyten, student engagement is described as a tendency to be involved in academic activities behaviorally, emotionally & cognitively. Studies have indicated that involving students in the procedure of learning helps increase their attention and focus and promotes meaningful learning experiences [12]. Engagement is also linked to improved student outcomes, such as improved grades and a decline in dropout rates [13]. Improved behavior of engagement results in increased academic self-confidence which further results in improved scores in examinations. This would also be of help to the potential learners. A positive impact is seen on the engagement and motivation of a learner in case the teacher enjoys what she is teaching, is confident, works toward academic growth and development of the learners, and can manage her own emotions to positively affect students' “motivation and engagement” in the classroom [14]. IIn the current study, the learning behavior of engagement showed significant improvement compared to teenage learners and adult learners.

Collaboration is a learning behavior of significance included in the participation-centered learning behaviors. This learning behavior plays a significant role in group activities. Research indicates that collaboration in learning leads to a deeper understanding of the concept and helps in the development of communicative skills and higher-order thinking. There is an increased interaction between learners and the positive outcomes of collaborative learning help to increase the self-esteem and responsibility of the learner [15]. The study by Scager et al., in 2016, dealt with five different life sciences undergraduate courses and factors that help in increased effectiveness of "collaboration." The results of this study suggest that positive interdependence amongst the group members during collaborative activities and the motivation and support extended to each other, make the collaboration meaningful and indicate strong self-regulatory processes [16]. The results of this study show that adult learners place a greater importance on the learning behavior known as "collaboration" than do adolescents. Studies indicate that there is a significant correlation between the learners’ academic achievement and communication. Communication in a classroom should be a two-way process, interactive in nature. Effective communication by the teacher is a precondition to effective learning. However, appropriate communication among the learners also plays an important role. Restructuring of the learning process and introduction of on-task verbal interaction between learners, help them to benefit from the more knowledgeable others in the group for enhancing their cognition and learning experience [17]. The present study showed learning behavior of communication was discovered to be significantly more valuable for adult learners than for teenagers in the current study. The learning behavior of responsiveness is a participation-centered learning behavior dealing with the quality of speedy and positive responses of the learners. According to research, a social interactive perspective’ has the social context of schooling as its thrust area and is based on the social constructivism views derived from the theories of Piaget and Vygotsky [18].

Limitations of the present study are the scope of this research is restricted to an “English medium CBSE school” and a “health professions institute” in Nagpur, India. The current study was conducted in groups of adolescent and adult students only. However, research conducted at the grade, age, person, and subject levels may be more useful in understanding learning behaviors and their transition in learners as opposed to the desired end. The future scope of the present study is research should be conducted to assess the usefulness of the developed “self-monitoring checklist” in instilling appropriate participation-centered learning behaviors in both adolescent and adult learners. Research needs to be undertaken so as to assess the usefulness of the generated “self-monitoring checklist” in instilling appropriate participation-centered learning behaviors in adolescent and adult learners. Creation of an “Android app” for the learners' convenience can be done. “Multi-centric studies” should be conducted to assess learning behaviors in various environments and age groups.

## Conclusions

The study findings indicate that gender-based differences in learning behaviors in both adolescent and adult learners are not substantial. The mean scores of participation-centered learning behaviors were notably greater among adult learners than in “adolescent learners.” The qualitative component's conclusions were consistent with the results of the learning behavior questionnaire examination. With the quantitative and qualitative analytical findings, a “self-monitoring checklist” for monitoring “learning behaviors” is constructed. Learning behavior is a complicated construct that cannot be described and appears to have arisen through the learner's triangle of interactions with "curriculum," "others," and "self" which includes parents, instructors, and classmates. Researchers have defined learning behaviors using characteristics such as involvement, cooperation, motivation, communication, engagement, and many more.
